# Met in Urological Cancers

**DOI:** 10.3390/cancers6042387

**Published:** 2014-12-16

**Authors:** Yasuyoshi Miyata, Akihiro Asai, Kensuke Mitsunari, Tomohiro Matsuo, Kojiro Ohba, Yasushi Mochizuki, Hideki Sakai

**Affiliations:** Department of Urology, Nagasaki University Hospital, 1-7-1 Sakamoto, Nagasaki 852-8501, Japan; E-Mails: rsjfk462@yahoo.co.jp (A.A.); kmitsunari@nagasaki-u.ac.jp (K.M.); tomo1228@nagasaki-u.ac.jp (T.M.); ohba-k@nagasaki-u.ac.jp (K.O.); mochi@nagasaki-u.ac.jp (Y.M.); hsakai@nagasaki-u.ac.jp (H.S.)

**Keywords:** Met, prostate cancer, renal cell carcinoma, urothelial cancer, pathological features, survival, target therapy

## Abstract

Met is a tyrosine kinase receptor that is considered to be a proto-oncogene. The hepatocyte growth factor (HGF)-Met signaling system plays an important role in tumor growth, invasion, and metastasis in many types of malignancies. Furthermore, Met expression has been reported to be a useful predictive biomarker for disease progression and patient survival in these malignancies. Many studies have focused on the clinical significance and prognostic role of Met in urological cancers, including prostate cancer (PCa), renal cell carcinoma (RCC), and urothelial cancer. Several preclinical studies and clinical trials are in progress. In this review, the current understanding of the pathological role of Met in cancer cell lines, its clinical significance in cancer tissues, and its predictive value in patients with urological cancers are summarized. In particular, Met-related malignant behavior in castration-resistant PCa and the different pathological roles Met plays in papillary RCC and other histological types of RCC are the subjects of focus. In addition, the pathological significance of phosphorylated Met in these cancers is shown. In recent years, Met has been recognized as a potential therapeutic target in various types of cancer; therapeutic strategies used by Met-targeted agents in urological cancers are summarized in this review.

## 1. Introduction

Met is a member of cell surface tyrosine kinase (TK) family that was isolated from a chemically transformed human malignant cell line in the 1980s [1]. Hepatocyte growth factor (HGF, also known as scatter factor) is the only endogenous ligand of MET, and HGF/Met signaling can regulate various cellular processes, such as proliferation, apoptosis, and migration [2,3]. Therefore, this signaling is important in physiological activities including maintenance of homeostasis and wound repair [3,4,5].

Since cell proliferation, migration, and angiogenesis are important steps in malignant cell dissemination, many investigators have paid special attention to the relationship between Met overexpression and malignant aggressiveness, including tumor invasion and metastasis. A recent review indicated that enhanced HGF/Met signaling is found in various types of cancers [6]. In addition, Met expression has been reported to be positively associated with tumor growth, invasion, and metastasis in cancer patients [7]. Indeed, our previous reports showed that Met played a crucial role in tumor growth, metastasis, and prognosis in patients with bladder cancer and renal cell carcinoma [8,9].

**Figure 1:**
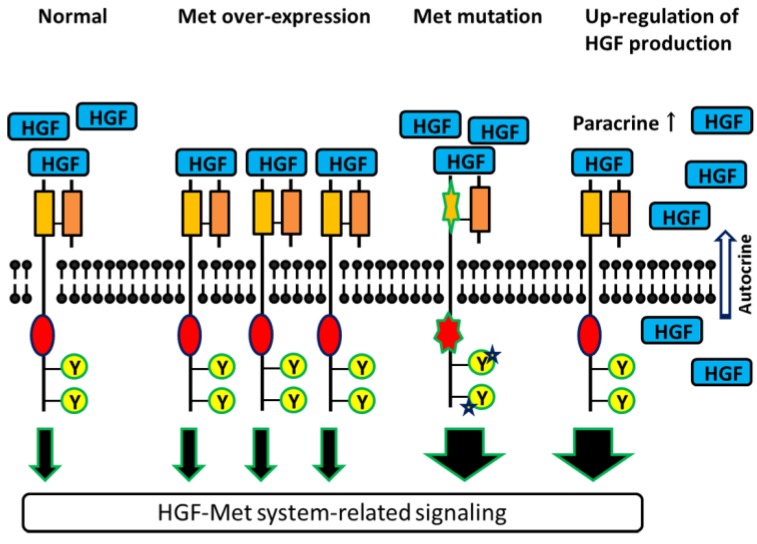
In cancer, HGF-Met signaling is up-regulated by various systems, including over-expression or mutation of Met and increased HGF production.

Many investigators have suggested that evaluation of the expression and biological activity of Met is important in understanding the pathological characteristics, malignant behavior, and strategies of observation and treatment for patients with malignancies. However, it should be noted that there is a wide range of variability in the quantification and pathological significance of Met in previous reports. In particular, variability has been demonstrated in the immunohistochemical technique most commonly used for evaluation of Met expression in animal and human tissues. Possible reasons that have been suggested for such discrepancies include differences in sample size, antibodies used, methods of evaluation, and condition of the tissue samples [8]. It has subsequently been suggested that quantification of phosphorylated Met could be a useful marker of Met signaling activity and Met-related pathological functions; this is discussed in greater detail below. Aberrant MET signaling is the cause of the pathological behavior and malignant aggressiveness of Met in human malignancies. As shown in Figure 1, it is regulated through various mechanisms.

The common mechanism of MET signaling activation is transcriptional deregulation, resulting in MET overexpression in malignant cells. In addition, *MET* gene mutation and amplification of the *MET* gene locus, resulting in receptor overexpression, are also characterized aberrancies. Mutation of the *MET* gene is thought to be a rare event in cancer, but it is often found in papillary renal cell carcinoma (RCC) and childhood hepatocellular carcinoma [9,10]. Conversely, *MET* gene copy number gain has been reported to be common and is associated with a poor prognosis in patients with lung cancer [11]. The fact that the activation and up-regulation mechanisms of Met in malignancies depend on the type of cancer and background of the patient should be noted when discussing the clinical significance of Met.

HGF-Met signaling has been reported to interact with various cancer-related molecules that promote tumor growth and metastasis. For example, HGF-Met signaling can promote angiogenesis though interaction with the vascular endothelial growth factor (VEGF) and VEGF receptor (VEGFR) signaling system [3,12]. In addition to VEGF, HGF-Met signaling has been known to mediate angiogenesis through regulation of thrombospondin (TSP)-1, which is a known anti-angiogenic factor [13]. Interestingly, HGF-Met signaling has been found to mediate angiogenesis through positive regulation of VEGF and negative regulation of TSP-1 [13]. Furthermore, Met is reported to be associated with cancer-related activities through various mechanisms including carcinogenesis, cell migration, and chemoresistance [14,15,16].

There are three different strategies to inhibit Met signaling: antagonism of receptor-ligand interaction; inhibition of the TK catalytic activity; and inhibition of the interaction between the receptor and intracellular signaling effectors. By understanding the detailed structure, function, and regulative mechanisms of HGF-Met signaling, it has been possible to develop various Met inhibitors (Table 1). At present, Met inhibitors are divided into two major classes: monoclonal antibodies that compete with HGF for binding to Met and Met TK inhibitors (TKIs). In Table 1, Met inhibitors for various types of malignancies are shown, including those for urological cancers; some of these drugs are in preclinical development and others are in or have completed clinical trials. However, as shown in Table 1, the number of Met-targeted therapies in urological cancer clinical trials is limited. Therefore, the pathological role, clinical significance, and predictive value of Met, and the clinical development of Met-targeted therapy in urological cancers, including PCa, RCC, and urothelial cancer, are reviewed in this manuscript.

**Table 1:** Met-targeting therapies for malignancies including urological cancers.

	Type of Malignancy	Urological Cancers (Phase)
Anti-Met mAbs		
LY-2875358	Lymphoma	
Onartuzumab	Breast, Colon, Lung, Stomach	
Small anti-MET TKIs		
Cabozantinib (XL 184)	Brain, Breast, Lung, Pancreas, Thyroid *	Prostate (II/III), Renal (II/III)
Crizotinib	Lung *, Lymphoma	
Foretinib (XL 880)	Breast, Head and neck, Lung, Stomach	Papillary renal (II)
Golvatinib (E7050)	Brain, Head and neck, Liver, Stomach	
MGCD 265	Lung	
Tivantinib (ARQ 197)	Breast, Colon, Liver, Lung, Myeloma	Prostate (II), Renal (I/II)

* Approved by U.S. Food and Drug Administration (PDA).

## 2. Prostate Cancer

PCa is the most common cancer in men. In the United States, it is estimated that 29,000 patients died from PCa in 2013 [17]. Numerous studies have investigated the pathological characteristics and cancer-related changes to the molecules and genes of PCa cells *in vivo* and *in vitro*. As mentioned above, the Met TK receptor has been shown to play a role in the proliferation and progression of many types of malignancy, including PCa. In this section, the pathological significance, prognostic roles, and possible therapeutic use of targeting Met in PCa are discussed. In addition, the pathological role of Met expression in castration-resistant PCa (CRPC) is shown.

### 2.1. The Function of Met Expression in Prostate Cancer Cell Lines

Numerous studies have demonstrated that in human PCa cell lines the MET signaling system plays an important role in cell survival by enhancing cell proliferation and suppressing apoptosis [18,19]. In addition, knockout of the Met gene inhibits tumor cell metastasis [20]. Recent studies have shown that the expression of the tumor suppressor miR-34 is inversely correlated with MET expression in PC-3 cells [21]. In addition, it has been suggested that miR-34 may inhibit the migration of PCa cells by regulating Met activity. Thus, several reported suggested that Met plays a critical role in the malignant aggressiveness of PCa cells.

In discussing the pathological mechanisms of Met in PCa, it is important to consider *in vivo* and *in vitro* androgen-sensitivity. PCa cells initially depend upon androgen stimulation, through androgen receptor signaling, for cell survival and malignant behavior. In fact, androgen deprivation is the standard treatment for patients with advanced PCa. However, as the cells become androgen-independent, they become more aggressive. Most recurrent and metastatic tumors that occur after androgen-deprivation therapy include castration-resistant PCa cells [22]. There is currently no curative therapy for CRPC, and patients tend to die due to invasion and metastasis-related pathological conditions.

Among the most widely used human PCa cell lines, MET protein is highly expressed in androgen receptor-negative cell lines (PC-3 and DU145) but only minimally expressed in androgen receptor-positive cell lines (LNCaP, LAPC-4, CWR22, and LUCaP) [23]. In these cell lines, the *MET* mRNA expression levels are reported to be similar to the protein levels. The androgen receptor-negative PCa cells, PC3 and DU145, have a higher malignant potential, including increased cell proliferation and migration, suppressed apoptosis, and a higher rate of angiogenesis when compared to androgen-receptor-positive cells. Based on these findings, it has been hypothesized that the loss of androgen receptor expression and increased Met expression contribute to the malignant potential of CRPC. Interestingly, continuous androgen-deprivation of the androgen-sensitive PCa cell line, LNCaP, results in decreased androgen receptor expression and increased Met expression [24]. In addition to these *in vitro* studies, the pathological activity of Met in CRPC has also been confirmed using CRPC cell lines and animal xenograft models [23,25]. From these findings, Met is speculated to be potential therapeutic target for CRPC. In fact, Met inhibitors have been shown to suppress cell proliferation and tumor growth of androgen receptor-negative (androgen-insensitive) PCa cell lines [26].

Furthermore, the importance of cancer stem cells (CSCs) in prostate cancer should be emphasized. According to the CSC hypothesis, tumor growth is sustained by a subpopulation of cancer stem/progenitor-like cells. Like other malignancies, human prostate cancer tissue contains neoplastic cells with self-renewal and clonogenic potential, which can be enriched and perpetuated in prostaspheres [27]. When MET is activated in prostate cancer cells, a stem-like phenotype is induced. MET is thought to regulate tumor infiltration in surrounding tissues by acquisition of a stem-like phenotype [28].

### 2.2. Expression/Activation of MET in Prostate Cancer and Correlation with Clinicopathological Characteristics

Some investigators have showed the relationships between Met expression and clinicopathological features in patients with prostate cancer [29,30,31,32,33,34]. We list a summary of their results as Table 2. In regard to Gleason score, several reports have indicated MET expression had a positive correlation [29,31,33,34]. On the other hand, there is little information regarding its correlation with TNM stage. Indeed, there is one study that indicates that the expression of MET is not significantly associated with tumor stage, nodal stage, or surgical margin status [32]. Furthermore, although MET expression in metastatic tumors is higher than in primary PCa, it is not a significant predictive factor in patients with a Gleason score of 6 or 7 [31]. Thus, further detailed studies are necessary to decisively determine the clinical significance and pathological role of MET in PCa patients.

**Table 2:** Relationship between Met expression and pathological features in prostate cancer.

Year	No. pts	Clinicopathological Features				Ref.
		High GS	High T Stage	Presence of LN Metastasis	Presence of Distant Metastasis	
1995	73	*p* < 0.01	–	–	–	[29]
1995	128	NS	–	–	*p* < 0.05	[30]
1999	36	*p* < 0.05	–	–	–	[31]
2002	86	NS	–	–	–	[32]
2004	66	*p* < 0.05	–	–	–	[33]
2013	3378	*p* < 0.01	NS	NS	–	[34]

No. pts; number of patients, GS; Gleason score, LN; lymph node, NS; not significance, Ref; reference.

For CRPC in particular, though, MET expression has been reported to be upregulated and is thought to play a role in angiogenesis, cell invasion, and metastasis to distant tissues [35]. As mentioned previously, *in vitro* studies have shown a significant correlation between increased Met expression and androgen-independent growth [23,24]. These *in vitro* and *in vivo* findings suggest that the expression of Met is correlated with the emergence of castration-resistant tumor growth, and that Met is a potential therapeutic target in CRPC.

### 2.3. MET-Targeting Therapies for Patients with Prostate Cancer

#### 2.3.1. Cabozantinib

Cabozantinib (XL184) is an orally bioavailable TKI with potent activity against MET and VEGFR2. Cabozantinib blocked the progression of osteolytic and osteoblastic lesions in a xenograft model of CRPC in bone [36]. A phase II, randomized, discontinuation trial has been conducted in patients with advanced PCa, showing that cabozantinib has clinical efficacy in men with CRPC [37]. The trial showed that cabozantinib led to a reduction in soft tissue lesions, improved progression-free survival (PFS), improved resolution of bone scans, decreased levels of bone turnover markers, less pain, and reduced narcotic use. Two phase III clinical trials were done in men with metastatic CRPC. The first is the Cabozantinib Met Inhibition CRPC Efficacy Trial (COMET)-1 (NCT01605227), which aims to evaluate the efficacy of cabozantinib compared to prednisone. In this study, the overall survival of men previously treated for metastatic CRPC with bone-dominant disease, who have experienced disease progression on docetaxel-containing chemotherapy, abiraterone or MDV3100, was assessed. The second study is the Cabozantinib Met Inhibition CRPC Efficacy Trial (COMET)-2 (NCT01522443). This study aims to evaluate the effect of cabozantinib *versus* mitoxantrone plus prednisone on pain response and bone scan response in men with CRPC. However, COMET-1 did not meet demonstrating a statistically significant increase in overall survival, and based on the outcome of COMET-1, enrollment in COMET-2 was halted.

#### 2.3.2. Tivantinib

Another small molecule inhibitor of Met, tivantinib (formerly ARQ 197), is being studied in PCa. A phase I trial of ARQ 197 was conducted to evaluate the safety of tivantinib. The study showed that tivantinib safely inhibited intratumoral c-MET signaling, but no responses, according to the Response Evaluation Criteria in Solid Tumors, were observed [38]. A phase II trial is ongoing in men with asymptomatic or minimally symptomatic metastatic CRPC (NCT01519414). The purpose of this trial is to investigate the efficacy of tivantinib compared to placebo for treating patients with metastatic PCa.

#### 2.3.3. Sorafenib

Sorafenib is a multikinase inhibitor targeting Raf kinase (serine-threonine kinase) and tyrosine kinases, including VEGFR, platelet-derived growth factor receptor (PDGFR), c-kit, c-Met. A phase II trial of sorafenib monotherapy for CRPC reported relative tolerance of this agent. However, although initially 2 of 22 patients treated with sorafenib showed evidence of improved bony metastatic lesions, most patients (21 of 22) had disease progression [39].

#### 2.3.4. Sunitinib

Sunitinib is an oral multitargeted inhibitor of VEGFR, PDGFR, c-kit, FLT-3, and c-Met. A phase III trial that evaluated angiogenesis-targeted sunitinib therapy in a randomized, double-blind trial of metastatic CRPC concluded that the addition of sunitinib to prednisone did not improve OS compared with placebo in docetaxel-refractory metastatic CRPC [40].

#### 2.3.5. Cause of Failure in MET-Targeting Therapies

As mentioned above, various types of MET inhibitors have been studied in clinical trials. However, unfortunately, almost of them showed “negative” results. As reason of such failure, several factors including study design, patient’s selection, and unexpected side effect are speculated. In this review, we emphasize the fact that such molecular-targeting therapies used for patients with CRPC. In addition, in many cases, CRPC patients were previously treated with docetaxel prior to such therapies. From these facts, we believe that more detailed information regarding pathological roles of Met expression in patients with CRPC are essential to improve the anti-tumor effect.

## 3. Renal Cell Carcinoma

Renal cell carcinoma (RCC) is the third most frequent cancer originating from the genitourinary organs. It is estimated that 65,000 patients were diagnosed with RCC in the United States in 2013 [17]. RCC originates from the proximal tubule of the kidney or the collecting duct and is classified into four major histological types: clear cell (conventional), papillary, chromophobe, and collecting duct. Among these, clear cell (conventional) RCC is the most common histological type and accounts for 75%–80% of all cases. Papillary RCC is the second most common histological type and accounts for 10%–15% of cases. Papillary RCC can be further divided into two morphological subtypes; type 1 consists of predominantly basophilic cells and type 2 consists of mostly eosinophilic cells. In recent years, cytogenetic and molecular studies have indicated that these different RCC pathological types possess different characteristics and respond differently to therapies. For example, papillary RCC is more frequent in male patients and tends to be of a low stage compared to clear cell or chromophobe RCC [41]. Therefore, it is necessary to discuss the pathological role of Met according to the different subtypes. Inactivation of the von Hippel-Lindau (*VHL*) gene should also be considered in this discussion of the pathological characteristics and molecular mechanisms of RCC.

### 3.1. The Function of Met Expression in Papillary RCC

The clinical significance and pathological role of MET has been most widely investigated, and is best understood, in papillary RCC. Several activating missense mutations of the *MET* gene have been described in individuals with papillary RCC [42]. Several mutations in the MET TK domain, both in the germline of hereditary papillary RCC families (M1131T, V1188L, D1228H, Y1230C) and in a subset of sporadic papillary RCC patients (L1195V, D1228H, Y1230H, M1250T), have been described [43]. Similar trends in hereditary papillary RCC have also been reported in another study [44]. Research on MET and papillary RCC reported the interaction between c-MET and VEGF; the development of resistance to VEGF-targeted therapy correlated with the up-regulation of c-MET expression. This result suggests that the acquired resistance to VEGF pathway inhibitors is frequently mediated by the activation of alternative signaling pathway (angiogenic escape), which induces c-MET overexpression [45].

### 3.2. The Function of Met Expression in Clear Cell RCC and Other RCCs

The most well-known form of inherited clear cell RCC is that associated with VHL syndrome. Inactivation of *VHL* gene function by mutation or methylation is found in patients with sporadic clear cell RCC, although it is rare in other histological RCC types. Several reports have found that Met protein is detectable in human clear cell RCC tissues [46,47,48,49]. VHL expression in RCC cells suppresses MET/β-catenin signaling, and a loss of VHL in conventional RCC has been shown to enable MET/β-catenin signaling *in vitro* [50]. Thus, it has been suggested that Met plays important roles in the carcinogenesis and malignant behavior of clear cell RCC via direct and indirect mechanisms.

### 3.3. Expression/Activation of MET in Renal Cell Carcinomas and Correlation with Clinicopathological Features

Strong expression of MET is observed in almost all papillary RCC and collecting duct carcinoma, however, it is uncommon in clear cell RCC and chromophobe RCC [48]. Gibney *et al.* have also reported that MET expression in papillary RCC and sarcomatous RCC is significantly higher than in clear cell RCC subtypes [51]. Thus, Met overexpression is speculated to be a characteristic of papillary RCC. It has been reported that MET expression is positively correlated with the nuclear grade, sarcomatoid component, and lymphatic invasion in patients with clear cell RCC [48]. Furthermore, the presence of phosphorylated MET is reported to be significantly correlated with malignancy aggressiveness and prognosis in clear cell RCC [52]. The clinical significance and prognostic role of MET in other types of RCC, including chromophobe and collecting duct carcinoma, is not fully understood. A summary of the correlations between MET expression and the clinicopathological features including malignant potential and survival of patients with RCC is shown in Table 3.

**Table 3:** Relationship between MET expression and grade, tumor stage, and prognosis for patients with renal cell carcinoma.

Year	No. pts	Pathological Features				Survival	Ref.
		High Grade	pT Stage	Lymph Node Metastasis	Distant Metastasis		
2006	114	NS	NS	NS	NS	NS	[52]
2006	96	*p* < 0.01	NS	NS	NS	-	[48]
2007	66	-	P < 0.01	-	*p* < 0.05	*p* < 0.01	[47]
2013	317	*p* < 0.01	-	-	-	*p* < 0.05	[51]

No. pts; number of patients, NS; not significant, Ref.; reference.

### 3.4. Met-Targeted Therapies for Patients with Renal Cell Carcinoma

#### 3.4.1. Foretinib (XL880)

The drug foretinib, formerly XL880, is a multi-targeted receptor TKI that targets the MET, VEGFR2, platelet derived growth factor receptor (PDGFR) β, Tie-2, recepteur d'origine nantais (RON), KIT, and FLT3 receptors. A phase I study of foretinib has been completed, and two out of four patients with papillary RCC showed a partial response (PR) [53]. A phase II study of foretinib in patients with papillary RCC was open-label and nonrandomized [54]. In this study, 74 patients were enrolled and an overall response rate of 13.5% with a median PFS of 9.3 months was demonstrated. The most frequent adverse events in patients with any grade RCC were fatigue, hypertension, gastrointestinal toxicities, and pulmonary emboli. Interestingly, among patients with germline *MET* mutations, five (50%) of ten experienced a PR, and the remaining five (50%) patients had stable disease as the best response. A PR was seen in only five (9%) of 57 patients without germline *MET* mutations. Further investigations are necessary to clarify these results.

#### 3.4.2. Tivantinib (ARQ197)

Tivantinib (ARQ197) is an inhibitor of Met receptor TK activity. *In vitro* studies indicate that tivantinib inhibits colony formation in ACHN and 769P cells [51]. Following completion of a phase I dose-finding study [55], phase II trials are ongoing in patients with metastatic papillary RCC (NCT00345423).

#### 3.4.3. Cabozantinib (XL184)

Recently, a phase I study of cabozantinib (XL184), a small molecule inhibitor of TKs including MET and VEGFR2, was performed [56]. The study enrolled 25 patients with metastatic clear cell RCC for whom standard therapy had failed. Common adverse events included fatigue, diarrhea, nausea, proteinuria, decreased appetite, palmar-plantar erythrodysesthesia, and vomiting. PR was reported in seven patients (28%) with a median PFS of 12.9 months and a median overall survival of 15.0 months. From these preliminary data, a phase II study randomizing patients to cabozantinib or sunitinib in the first-line setting for mRCC has been initiated [45]. In papillary RCC, a special role for c-MET inhibitors may emerge because of the critical role of activating c-MET mutations [46].

## 4. Urothelial Cancer

Urothelial carcinoma (UC), especially bladder cancer, is the second most common genitourinary malignancy; it accounts for almost 5% of all newly diagnosed cancers. It is estimated that 72,570 patients were newly diagnosed with UC of the bladder in the United States in 2013. More than 90% of cases pathologically originate from urothelial cells. Papillary UCs are typically superficial at first presentation and are often multifocal. These tumors recur with a high frequency (>60%), and 10%–15% of them will progress to life-threatening malignancies over a longer period of time [17,57]. Several risk factors can be used to predict tumor progression, including histological grading, stage classification, tumor morphology, and the size and number of tumors [58,59,60,61,62,63]. The number of tumors and their classification stage are considered the most important prognostic factors for recurrence [59,60,61,62,63,64]. Patients with invasive and/or metastatic lesions have a poor prognosis. Tumor invasion and metastasis are regulated by complex mechanisms, including tumor growth, degeneration of stromal tissues, cell migration, and angiogenesis. Moreover, bladder cancer is characterized by distinct molecular characteristics. For example, FGR3 mutations are associated with low-stage and low-grade tumors, whereas p53 mutations are associated with high-stage and high-grade tumors [65,66]. Therefore, detailed information regarding these mechanisms is essential to formulate appropriate treatment and observation strategies for patients with UC [67]. In addition to MET expression, interaction with the Axl and PDGFR-α pathways contributes to bladder cancer progression [68]. RON, a macrophage-stimulating protein, is a distinct receptor TK in the Met proto-oncogene family [69]. Co-expression of RON and MET is significantly associated with decreased overall and metastasis-free survival [70]. RON-associated signaling may also play an important role in the progression of human bladder cancer. Some researchers have suggested that cross-talk exists between the RON and Met pathways [71,72]. Evaluation of RON and Met expression status may help to identify a subset of bladder cancer patients who require more intensive treatment.

### 4.1. Function of Met Expression in UC

In UC cell lines, Met mRNA has been detected by northern blot analysis [73] and reverse transcriptase polymerase chain reaction [74]. In the absence of point mutations, the expression of Met tends to positively correlate with cancer cell line differentiation [59]. However, since most of these studies have been *in vitro*, a detailed understanding of the pathological significance and regulative mechanism of MET in UC cells has not been achieved.

### 4.2. Expression/Activation of Met in UC and Correlation with Clinicopathological Characteristics

It was initially reported that Met expression could not be detected in the normal urothelium or in cases of UC of the kidney [75]. However, more recently, Met expression in urothelial cells, endothelial cells, and smooth muscle cells of the urinary organs has been detected [76]. A previous study had reported that the expression of Met in UC cells of the urinary bladder was positively associated with histological grading, non-papillary contour, tumor size, and muscle invasion [77]. In another study, antibody arrays were used to demonstrate that MET was strongly expressed in high-grade cases of bladder cancer [78]. However, some researchers have found contradictory results (Table 4). Thus, there is no general agreement on the relationship between MET expression and pathological features in patients with UC. However, in recent years, the consensus is that Met may play an important role in the carcinogenesis and malignancy aggressiveness in patients with UC [76,79,80]. Strong MET expression has been demonstrated in an invasive phenotype of bladder cancer [76], although varying levels of MET immunostaining are consistently detected in UC of the bladder [59]. In regard to correlation between MET expression and outcome in patients with bladder cancer, several reports have indicated that MET levels do correlate with the progression and several studies have shown that its overexpression is associated with shortened metastasis-free and overall survival in patients with bladder cancer [68,77,78]. These pathological significances and predictive roles of MET expression were showed in Table 4.

**Table 4:** Relationship between MET expression and pathological features and survival in urothelial carcinoma.

Year	No. pts	Clinicopathological Features				Outcome		Ref.
		Grade	T Stage	N Stage	M Stage	PD	Survival	
1998	49	NS	NS	NS	-		NS	[79]
2002	142	*p* < 0.01	*p* < 0.01	*p* < 0.01	-	*p* < 0.01	*p* < 0.05	[59]
2005	183	*p* < 0.01	*p* < 0.01	–	-	NS	*p* < 0.05	[69]
2006	173	*p* < 0.01	*p* < 0.01	*p* < 0.01	-		*p* < 0.05	[78]
2009	133	*p* < 0.01	*p* < 0.01	*p* = 0.01		NS	NS	[52]
2011	75	NS	NS	NS	NS		*p* < 0.01	[68]

No. pts; number of patients, PD, progression of disease, Ref; reference, NS; not significant.

The biological activities of Met are exerted by phosphorylation, and phosphorylation of Met is essential for the stimulation of downstream signaling pathways. For this reason, Miyata *et al.* [52] investigated the clinical significance and pathological function of MET phosphorylation in human bladder cancer tissues and found that MET expression was not a significant predictor of metastasis-free or cause-specific survival. However, phosphorylated MET was significantly associated with tumor aggressiveness and prognosis in patients with bladder cancer patients. In addition, among the different MET phosphorylation sites, phosphorylation of Y1349 was thought to play an important role in metastasis and survival. Phospho-Y1349 MET was correlated with the expression of matrix metalloproteinase-2 and -7, and E-cadherin [52].

### 4.3. Met-Targeted Therapy for Patients with Urothelial Carcinoma

Based on the pathological significance and prognostic role of MET in UC cells, many investigators have suggested that Met is a potential novel therapeutic target for these patients. SU-11274, a selective MET kinase inhibitor, is reported to affect the growth of bladder cancer [81]. In addition, several reports have shown that tumor growth and invasion by UC is inhibited by regulation of the HGF-Met signaling system [82,83]. However, to our knowledge, no clinical trial using a Met inhibitor is ongoing at present. Furthermore, a combination of TKIs could induce a synergistic antitumor effect, and improve therapeutic efficacy [84]. In the future, detection of Met co-expression molecules, combined with more selective, or multi-targeting, TKIs could enable individualized therapy in UC.

## 5. Conclusions

In this review, we have summarized the pathological role, clinical significance, and predictive value of Met in urological cancer *in vivo* and *in vitro*. In addition, we have reviewed the preclinical studies and clinical trials of Met-targeted therapies in patients with urological cancers. Numerous investigators have focused on the functions of Met at a genetic, molecular, and clinical level. However, much is still unknown. Based on previous reports and current studies, we believe that Met has the potential to be used as a predictive factor and therapeutic target in patients with urological cancers.
